# Enhancing survival outcomes in unresectable hepatocellular carcinoma: a prospective cohort study on the effects of Huaier granules with targeted therapy plus immunotherapy

**DOI:** 10.3389/fphar.2025.1529010

**Published:** 2025-03-27

**Authors:** Hui Li, Hongliang Zhang, Wenting He

**Affiliations:** ^1^ Affiliated Tumor Hospital of Xinjiang Medical University, Urumqi, China; ^2^ Department of Oncology, Traditional Chinese Medicine Hospital of Xinjiang Uygur Autonomous Region, Urumqi, China

**Keywords:** hepatocellular carcinoma, Huaier granules, immune checkpoint inhibitors, targeted therapy, clinical efficacy

## Abstract

**Objective:**

This study evaluated the clinical efficacy of Huaier granules combined with targeted therapy plus immunotherapy in patients with unresectable hepatocellular carcinoma (HCC) who had not undergone systemic treatment.

**Methods:**

Patients with unresectable HCC and no prior treatments were recruited from the Hospital of Traditional Chinese Medicine of Xinjiang and the First Affiliated Hospital of Xinjiang Medical University between March 2022 and July 2023. Patients received targeted therapy and immunotherapy with (exposure group) or without Huaier Granules (non-exposure group). The primary endpoint was progression-free survival (PFS), with secondary endpoints including 6-month PFS rate, HCC Symptom Severity Quantitative Response, EORTC QLQ-HCC18 Score, and safety.

**Results:**

The mPFS in the exposure group was 8.9 months compared to 5 months in the non-exposure group (P = 0.001; HR = 0.50). The 6-month PFS rates were 66.7% and 34.1% for the exposure and non-exposure groups, respectively (P = 0.001). The clinical efficacy rate of TCM symptom classification in HCC was higher in the exposure group (87.50% vs 59.09%; P = 0.001). The exposure group also showed improvement in fatigue (P = 0.023). Extrahepatic metastasis was an independent prognostic factor (HR = 1.77; P = 0.016), while Huaier granules reduced the risk of disease progression by 47% (HR = 0.53; P = 0.006). No significant differences were observed for adverse events. The most common adverse events were hypertension, proteinuria, abnormal liver function, and diarrhea.

**Conclusion:**

Huaier granules significantly prolong PFS and improve the 6-month PFS rate, reducing disease progression risk in HCC patients. Subgroup analysis showed more pronounced benefits in patients with vascular invasion and alcohol consumption, with mPFS extending beyond 1 year.

## Introduction

HCC is the predominant pathological type of primary liver cancer, accounting for 75%–85% of cases (Petrick et al., 2020). According to statistics from 2022, the detection rate and mortality rate of HCC in China rank fourth and second among all malignant tumors, respectively, with new and fatal cases representing nearly half of the global total (Xia et al., 2022). The 5-year survival rate is merely 12.1%, reflecting a high incidence and poor prognosis that contribute to significant disease and economic burdens (Wang and Deng, 2023). The incidence continues to rise, and it is estimated that by 2040, there will be 1.4 million new cases globally, presenting a persistent challenge to global public health (Rumgay et al., 2022).

In China, since more than 70% of patients have no chance of receiving curative treatment at the time of initial diagnosis and require systemic therapy, the current guidelines recommend a combination of targeted therapy and immunotherapy as the first-line treatment for patients with unresectable HCC. Although this approach has improved overall survival, the PFS is around 4–6 months, highlighting an urgent need for new strategies to improve PFS. Huaier granules have been shown to reduce the postoperative recurrence rate of liver cancer by 33% and are an independent protective factor for 5-year survival (*P* < 0.0001) (Chen et al., 2018; Liu et al., 2019). The Chinese Society of Clinical Oncology (CSCO) Primary Liver Cancer Diagnosis and Treatment Guidelines 2022 Edition provides a grade II recommendation for its use in postoperative adjuvant therapy. The Chinese Integrative Therapy of Primary Liver Cancer Working Group has recommended that, following the eight principles of Zheng identification, liver cancer should be categorized according to eight basic Zheng types: qi stagnation, blood stasis, heat, (water) dampness, qi deficiency, blood deficiency, yin deficiency and yang deficiency, after retrieving, organizing and reviewing both classical and modern TCM literature, summarizing a large amount of clinical experience and examining epidemiological survey results (Ling et al., 2018). In this study, we enrolled patients who were diagnosed with Qi Deficiency and Blood Stasis Syndrome, a Traditional Chinese Medicine (TCM) classification.

It has been demonstrated that adjuvant Huaier therapy Huaier can significantly prolongs patients survival as well as improving the quality of life for patients (Shi et al., 2023). However, due to a lack of large-scale, high-level clinical studies, no explicit recommendation has been made in the guidelines. Huaier granules may have a synergistic effect with targeted therapies and immune checkpoint inhibitors (ICIs), but there is currently no evidence from clinical studies to support this. Therefore, we conducted a prospective cohort study to observe the clinical efficacy of combining Huaier granules with targeted therapy and ICIs in patients with unresectable HCC who had not received systemic treatment.

## Material and methods

### Study population

Patients with unresectable hepatocellular carcinoma (HCC) who had not received prior anti-tumor systemic treatment were enrolled between 1 March 2022, and 1 July 2023, at the Hospital of Traditional Chinese Medicine of Xinjiang Uygur Autonomous Region and the First Affiliated Hospital of Xinjiang Medical University. This study is registered with the Chinese Clinical Trial Registry, registration number ChiCTR2400079626, and has been approved by the Ethics Committee of the Hospital of Traditional Chinese Medicine of Xinjiang Uygur Autonomous Region (2023-GS012). Inclusion criteria: 1) Age ≥18 years; 2) unresectable locally advanced, or metastatic HCC at diagnosis, (BCLC C or unsuitable for curative surgery or local treatment at stage B); 3) Presence of measurable lesions as assessed by the Response Evaluation Criteria in Solid Tumors (RECIST) version 1.1; 4) Performance status (PS) score of 0–1 and Child-Pugh liver function score ≤7; 5) Expected survival time of more than 12 weeks; 6) received targeted therapy plus immunotherapy 7) diagnosed with Qi Deficiency and Blood Stasis Syndrome, a Traditional Chinese Medicine (TCM) classification. The TCM syndrome diagnostic criteria for Qi Deficiency and Blood Stasis Syndrome referred to the “Guiding Principles for Clinical Research of New Chinese Medicine (Trial)” (Zheng, 2002) and the “Study of a qualitative diagnostic criterion for basic syndromes of traditional Chinese medicine in patients with primary liver cancer” (Ling et al., 2005). Main Symptoms: ① Fatigue and weakness ② Lumps under the costal cartilage; Other Symptoms: ① Dull complexion or cyan and purple lips and nails ② Numbness or abdominal distension after eating ③ Pain fixed without moving; Tongue Manifestation: light and fat tongue, or purple tongue or ecchymosis; Pulse Manifestation: Weak or thin or astringent pulse. If both main symptoms ① and ② are present along with at least two or more other symptoms, a diagnosis of Qi Deficiency with Blood Stasis Syndrome can be made. This syndrome type is an indication for Huaier Granules. Exclusion criteria: 1) History of autoimmune diseases or liver transplantation; 2) Acute or chronic active hepatitis B virus (HBV) or hepatitis C virus (HCV) infection, with HBV DNA levels greater than 2,000 IU/mL or 1,054 copies/mL, HCV RNA levels greater than 10^3^ copies/mL, or simultaneous positivity for hepatitis B surface antigen and anti-HCV antibodies (nucleotide antiviral therapy is permitted); 3) Central nervous system metastasis or presence of inferior vena cava tumor thrombus; 4) Interstitial lung disease or pulmonary fibrosis.

### Treatments

The use of Huaier granules during treatment was considered as the exposure factor, dividing the patients into two groups: those receiving Huaier granules (exposure group) and those who did not (non-exposure group). For the exposure group: Initiation of HuaiEr Granules oral administration concurrently with ICIs and targeted therapy treatments, 20 g per granule, produced and supplied by Qidong Gaitianli Pharmaceutical Co., Ltd., at a dosage of 20 g per administration, three times daily. Exposure to Huaier granules must be maintained for at least 15 days, and targeted therapy must include a minimum of two treatment cycles. For the non-exposure group, the dosage of ICIs and targeted drugs is to be determined by the attending clinician. No other systemic anti-tumor treatments are allowed for the target lesions, but localized palliative radiotherapy for isolated lesions (non-liver lesions) and treatment for bone metastases are permitted to control symptoms.

The use of other Chinese patent medicines or herbal decoctions with confirmed anti-tumor effects is prohibited. Following enrollment, patients will undergo baseline abdominal CT or MRI scans, with follow-up scans conducted every 1.5–2 months. Traditional Chinese Medicine (TCM) symptom grading for HCC and HCC-related quality of life assessments will be conducted at baseline and 1 day prior to the third treatment cycle. Before each treatment cycle, alpha-fetoprotein (AFP) levels and safety parameters will be monitored, and adverse events, as well as concomitant medication use, will be recorded until disease progression, patient death, or the study’s conclusion.

### Endpoints and assessments

The primary endpoint of this study was PFS. PFS was defined as the time from the date of enrollment to the occurrence of either disease progression, death, or the end of the study. The key secondary endpoints included 6-month PFS rate, HCC Symptom Severity Quantitative Response, EORTC QLQ-HCC18 Score and safety. The formula of Six-month PFS Rate is: (Number of non-progressed patients/Total number of patients) × 100%. Improvement Rate of HCC-Related Quality of Life: Quality of life is assessed across eight dimensions, each with a maximum score of 100 points. Scores are recorded at baseline and 1 day before the third treatment cycle. For each dimension, the scores from these two time points are compared; a decrease in score compared to the baseline is considered an improvement, while unchanged or increased scores indicate no improvement. The improvement rate is calculated for each dimension.

### Statistical analysis

The sample size for the cohort study was calculated using the formula:

$$n={\left(\frac{Z_{\alpha \;}\sqrt{2P\;\left(1-P\right)\;}+Z_{\beta }\sqrt{P_{1}\left(1-P1\right)+P_{2}\left(1-P2\right)}}{P_{1}-P_{2}}\right)}^{2}$$

Utilizing PASS 15.0 software, with a significance level (α) set at 0.05 and statistical power at 0.8 and based on literature and pre-exposure group results with P1 = 0.65 and P2 = 0.90, the calculation determined that n1 = n2 = 44. Accounting for a 10% loss to follow-up rate, the required sample size for each group was adjusted to 48, resulting in a total of 96 participants across the two groups.

The final follow-up date for this study is 30 October 2023. The PFS of the two groups was compared using SAS JMP (Pro 16.0) software for data processing. Continuous data with a normal distribution are presented as mean ± standard deviation, while data not following a normal distribution are expressed as median (interquartile range) [M (P25, P75)]. Baseline characteristics, including age, gender, alcohol consumption, BCLC stage, tumor number, presence of hepatitis, liver cirrhosis, intrahepatic metastasis, extrahepatic metastasis, vascular invasion, and AFP levels, were analyzed using the Chi-square test. For variables where the sample size did not meet the requirements of the Chi-square test, Fisher’s exact test was applied.

PFS was assessed using Kaplan-Meier survival analysis to construct survival curves. Subsequently, a multivariate Cox proportional hazards analysis was employed to explore further and establish a nomogram prediction model. The receiver operating characteristic (ROC) curve was plotted, and the area under the curve (AUC) was calculated. The model’s goodness-of-fit was evaluated using the Hosmer-Lemeshow test, with calibration curves constructed accordingly. Decision curve analysis (DCA) was also utilized to predict the risk of PFS events. The 6-month PFS rate was analyzed using the Chi-square test, with a significance level set at α = 0.05. A p-value of less than 0.05 was considered statistically significant.

## Results

### Baseline characteristics

**FIGURE 1:**
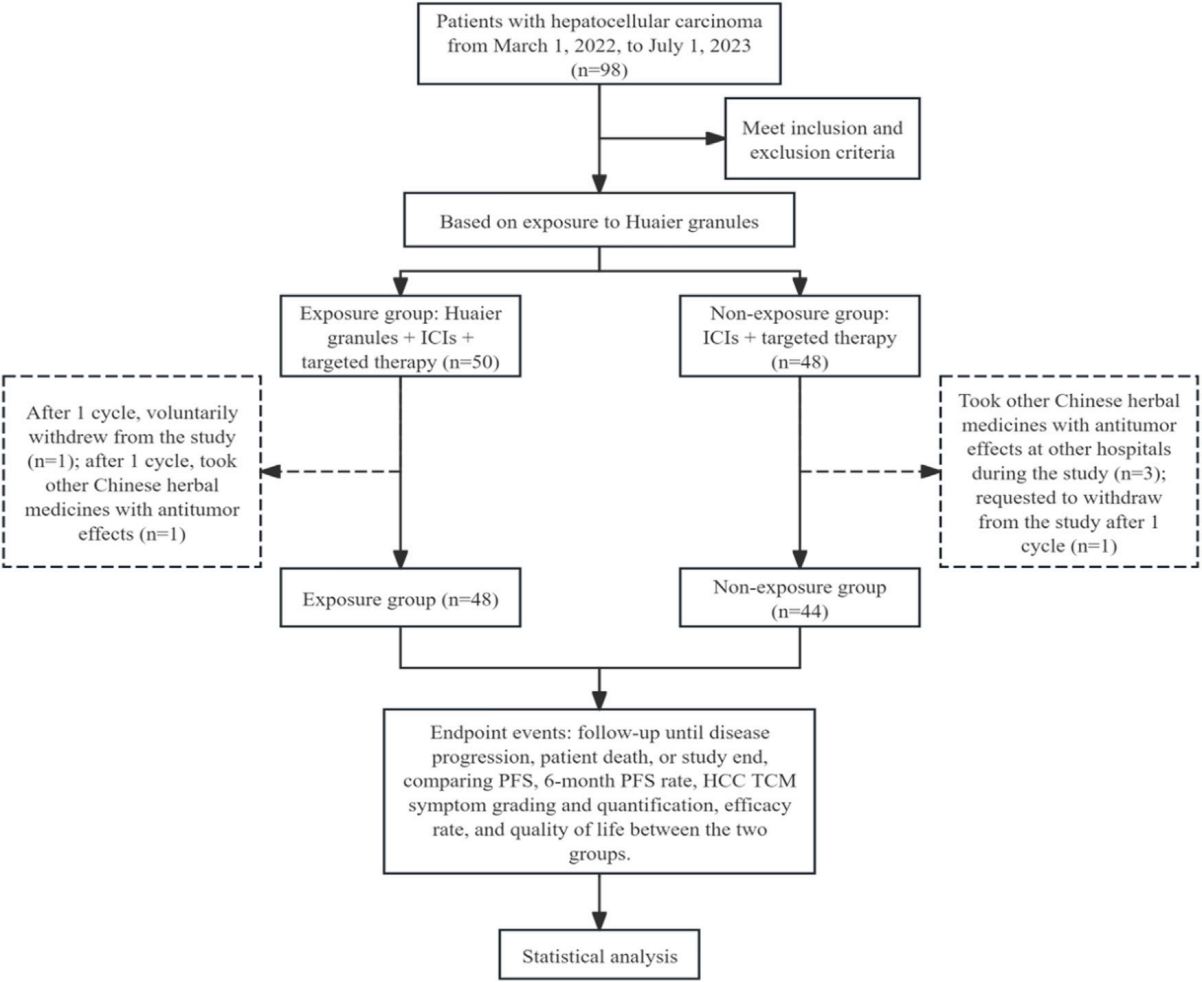
Flowchart of patient selection.

A total of 98 participants were initially enrolled in the study. During the treatment phase, six participants were excluded: four due to the use of additional anti-tumor herbal decoctions and two who voluntarily withdrew (Figure 1). Consequently, 92 participants were included in the final efficacy analysis—48 in the exposure group and 44 in the non-exposure group. The study concluded with the final follow-up on 30 October 2023.

Baseline characteristics of the participants are summarized in Table 1. At enrollment, the two groups were balanced and comparable regarding baseline characteristics such as gender, history of alcohol consumption, history of hepatitis, AFP levels, lesion size, presence of cirrhosis, intrahepatic and extrahepatic metastasis, tumor number, vascular invasion, Child-Pugh score, and BCLC staging. The average age of the exposure group was significantly higher than that of the non-exposure group (*P =* 0.009), but this bias does not exaggerate the efficacy of the exposure group.

**TABLE 1:** Baseline characteristics.

		Exposure group (n = 48)	Non-exposure Group (n = 44)	*χ* ^*2*^/Fisher’s exact test	*P*
Gender	Male	40 (83.33%)	36 (81.82%)	0.037	0.848
	Female	8 (16.67%)	8 (18.18%)		
Drinking	Yes	15 (31.25%)	21 (47.73%)	2.626	0.105
	No	33 (68.75%)	23 (52.27%)		
History of Hepatitis	Yes	40 (83.33%)	41 (93.18%)	-	0.202^#^
	No	8 (16.67%)	3 (6.82%)		
AFP (ng/mL)	<400	34 (70.83%)	25 (56.82%)	1.965	0.161
	≥400	14 (29.17%)	19 (43.18%)		
Tumor	<5 cm	20 (41.67%)	15 (34.09%)	0.560	0.454
	≥5 cm	28 (58.33%)	29 (65.91%)		
Cirrhosis	Yes	23 (47.92%)	25 (56.82%)	0.730	0.392
	No	25 (52.08%)	19 (43.18%)		
Intrahepatic Metastasis	Yes	30 (62.50%)	25 (56.82%)	0.308	0.578
	No	18 (37.50%)	19 (43.18%)		
Extrahepatic Metastasis	Yes	15 (31.25%)	22 (50.00%)	3.373	0.066
	No	33 (68.75%)	22 (50.00%)		
Child-Pugh Score	A	38 (79.19%)	32 (72.73%)	0.523	0.569
	B	10 (20.83%)	12 (27.27%)		
Tumor Count	Single	8 (16.67%)	6 (13.64%)	0.164	0.685
	Multiple	40 (83.33%)	38 (86.36%)		
Vascular Invasion	Yes	19 (39.58%)	20 (45.45%)	0.324	0.569
	No	29 (60.42%)	24 (54.55%)		
BCLC Stage	B	3 (6.25%)	8 (18.18%)	-	0.074^#^
	C	45 (93.75%)	36 (81.82%)		
Age	-	48 (61.89 ± 1.38)	44 (56.52 ± 1.44)	9.339	0.009^*^

Note: * indicates that there is a statistically significant difference between the two groups with *P* < 0.05 as determined by the Chi-square test. # indicates that Fisher’s exact test was used.

### Survival outcomes

**FIGURE 2:**
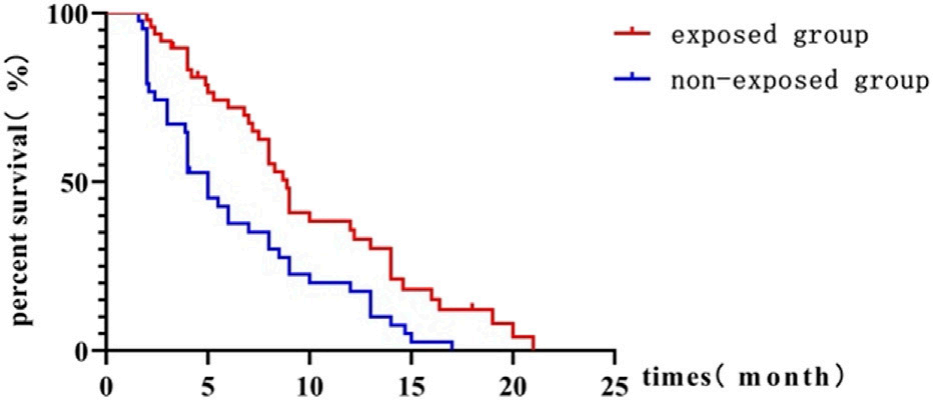
Kaplan-Meier curve comparing progression-free survival between exposed and non-exposed groups.

The exposure group had a significantly longer mPFS of 8.9 months compared to 5 months in the non-exposure group (HR = 0.50; 95% CI, 0.317–0.783; P = 0.001) as shown in Figure 2. The 6-month PFS rate was significantly higher in the exposure group compared to the non-exposure group, at 66.7% versus 34.1%, respectively, with a statistically significant difference (*P =* 0.001).

Subgroup analysis suggested that those with hepatitis, cirrhosis, vascular invasion, extrahepatic metastasis, tumor maximum diameter ≥5 cm, a history of alcohol consumption, no history of alcohol consumption or baseline AFP ≥400 ng/mL, had the prolonged mPFS in the exposure group compared to the non-exposure group, with all differences being statistically significant. Detailed results are presented in Table 2 and Supplementary Figure 1.

**TABLE 2:** Subgroup analysis of PFS between two groups.

Subgroup		PFS (95%CI)		Chi-square value (*χ* ^*2*^)	*P*
		Exposure group	Non-exposure group		
Hepatitis		9 (7.2.13)	5 (4.8)	9.3988	0.0022*
Cirrhosis		10 (5.14)	4 (3.6)	8.8167	0.0030*
Vascular invasion	Yes	13 (6.20)	3 (2.7)	12.8767	0.0003*
	No	8.3 (6.8.9)	6 (4.10)	0.9702	0.3246
Drinking	Yes	14 (7.19)	5 (2.9)	6.6855	0.0097*
	No	8.7 (5.3.9)	5 (3.8)	5.2260	0.0223*
Tumor size	≥5 cm	9 (6.14)	4 (2.1.6)	12.2333	0.0005*
	<5 cm	8 (4.2.12)	8 (4.13)	0.8193	0.3564
Sintilimab plus bevacizumab	Yes	6.8 (4.10)	5 (2.1.8)	2.0846	0.1488
	No	9 (7.5.14)	5 (3.9)	7.3017	0.0069*
Sintilimab	Yes	9 (7.5.13)	5 (3.10)	3.3245	0.0683
AFP level (ng/mL)	<400	8.9 (7.2.12.2)	6 (4.8.5)	5.0665	0.0244*
	≥400	9 (4.14.6)	3 (2.8)	5.7314	0.0167*
Extrahepatic metastasis	Yes	8.3 (4.12.2)	4 (2.5)	5.996	0.014*
	No	9 (6.8.14)	8 (3.9.10)	3.641	0.056

*Indicates a statistically significant difference between the two groups.

In patients with hepatitis, cirrhosis, vascular invasion, extrahepatic metastasis, tumor diameter ≥5 cm, the mPFS was significantly extended in the exposure group compared to the non-exposure group (9 months vs 5 months, 10 months vs 4 months, 13 months vs 3 months, 8.3 months vs 4 months, 9 months vs 4 months, and 9 months vs 5 months, respectively), all showing statistical significance (*P =* 0.002, *P =* 0.003, *P =* 0.000, *P =* 0.014, *P =* 0.001, and *P =* 0.007) (Supplementary Figure 1). In patients with a history of alcohol consumption, mPFS was extended by 9 months in the exposure group compared to the non-exposure group (14 months vs 5 months; *P =* 0.010), while in those without a history of alcohol consumption, mPFS was extended by 3.7 months (8.7 months vs 5 months; *P =* 0.022). For patients with baseline AFP ≥400 ng/mL, mPFS was extended by 6 months (9 months vs 3 months; *P =* 0.017), and for those with baseline AFP <400 ng/mL, mPFS was extended by 2.9 months (8.9 months vs 6 months; *P =* 0.024). All comparisons were statistically significant Supplementary Figure 1.

### Safety results and liver cancer-related quality of life

The most common adverse events of any grade observed were primarily hypertension, proteinuria, abnormal liver function, diarrhea, and hypothyroidism (Table 3). All of them were of graded 1 or 2, indicating a favorable safety profile. No statistically significant difference was observed between the two groups.

**TABLE 3:** Incidence of Adverse events.

	Exposure group (n = 48)	Non-exposure group (n = 44)	*χ* ^*2*^/Fisher’s exact test	*P*
Hypertension	3 (6.25%)	7 (15.91%)	-	0.133^#^
Proteinuria	15 (31.25%)	8 (18.18%)	2.121	0.145*
Abnormal Liver Function (Baseline Normal, Post-Treatment Abnormal)	15 (31.25%)	7 (15.91%)	3.032	0.082
Diarrhea	13 (27.08%)	9 (20.45%)	0.557	0.455
Hypothyroidism	18 (37.5%)	19 (43.18%)	0.308	0.579

*Chi-square test, *P* > 0.05 indicates no statistical significance.

^#^Fisher’s Exact Test.

This study utilized the EORTC QLQ-HCC18 scoring scale, which includes eight dimensions of quality of life related to liver cancer. Only the fatigue dimension showed a statistically significant improvement, with improvement rates of 68.75% in the exposure group versus 45.45% in the non-exposure group (*P =* 0.023).

### COX regression model analysis

A multivariate COX proportional hazards regression model was used to explore factors influencing the prognosis of all HCC patients enrolled. PFS was used as the dependent variable, and independent variables included the presence of extrahepatic metastasis, vascular invasion, AFP ≥400 ng/mL, and treatments. Analysis indicated that extrahepatic metastasis was an independent prognostic factor, with the risk of disease progression being 1.807 times higher in patients with extrahepatic metastasis compared to those without (*HR =* 1.807; 95% *CI*, 1.138–2.867; *P =* 0.013). The use of Huaier Granule was identified as a protective factor (*HR =* 0.514; 95% *CI,* 0.326–0.810; *P =* 0.004) as shown in Table 4.

**TABLE 4:** Multivariate cox analysis of factors influencing disease progression.

Factor	β	SE	Wald *χ* ^*2*^	*P*	HR	95%CI
Extrahepatic metastasis (Yes/No)	0.296	0.118	6.152	0.013	1.807	1.138–2.867
Huaier granule (Yes/No)	0.333	0.116	8.217	0.004	0.514	0.326–0.810

### Construction of a nomogram prediction model

**FIGURE 3:**
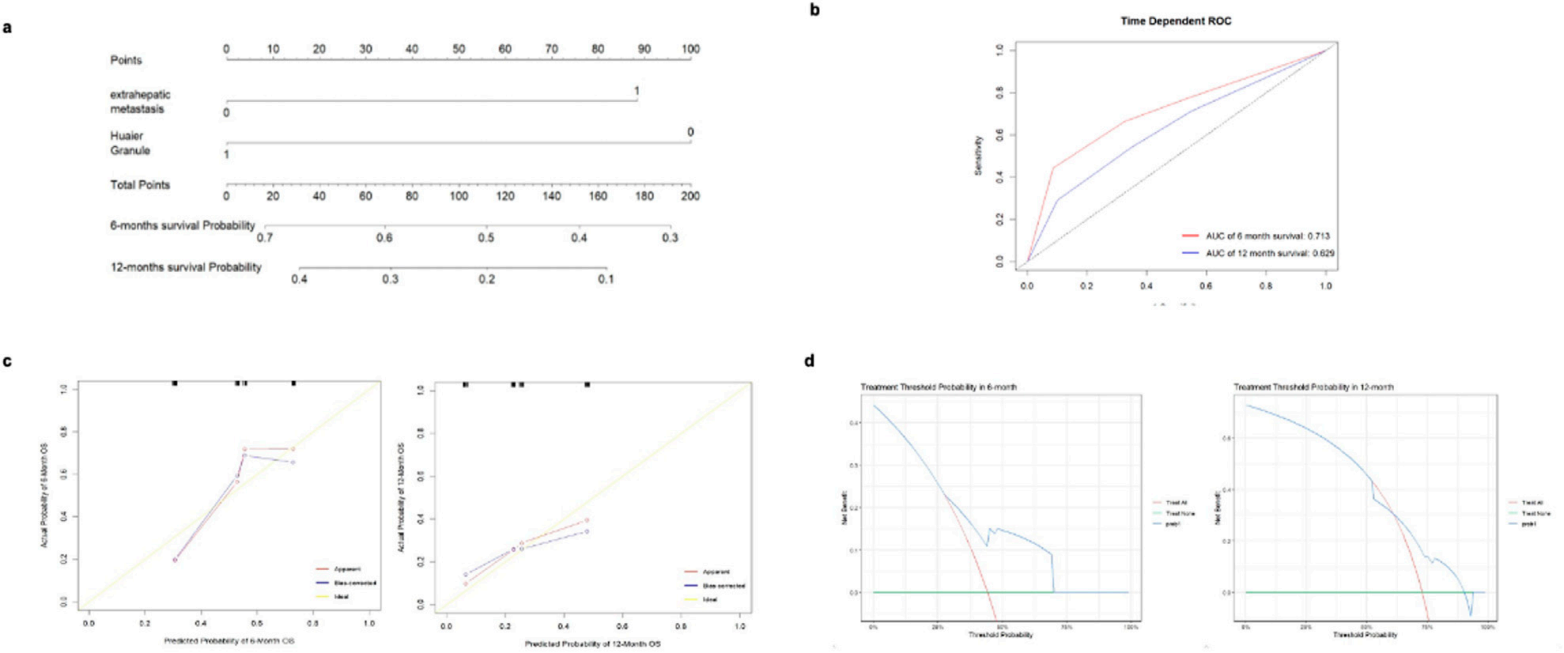
Analysis of predictive accuracy for 6 and 12-month survival probabilities. **(A)** nomogram for predicting the risk of PFS events. **(B)** ROC curve for predicting the risk of PFS events using the cox proportional hazards model. **(C)** Calibration curve of the nomogram for predicting the risk of PFS events. **(D)** Decision curve for the nomogram prediction model of PFS events.

Based on the results of the multivariate Cox proportional hazards analysis, exposure to Huaier Granule and the presence of extrahepatic metastasis were identified as key factors for inclusion in the nomogram prediction model. The nomogram was developed (Figure 3A), with each variable assigned a specific score. By adding up these scores, the probability of PFS events for individual patients can be estimated, with a higher total score indicating a greater likelihood of PFS events.

### Performance and clinical utility of the predictive model

The ROC curve was generated to evaluate the predictive accuracy of the nomogram. The area under the curve (AUC) for the 6-month follow-up prediction model was 0.713, while the AUC for the 12-month follow-up prediction model was 0.629 (Figure 3B). At the 6-month follow-up, the calibration curve of the nomogram demonstrated good concordance between predicted and observed outcomes (Figure 3C). The Hosmer-Lemeshow goodness-of-fit test indicated that the model fit was not statistically significant (P > 0.05), suggesting a good alignment with the observed data. The 12-month follow-up exhibited similar results, though the model’s fit was superior at 6 months compared to 12 months. Decision curve analysis (DCA) was conducted using the nomogram prediction model to evaluate PFS events for the selected variables. The analysis showed that at the 6-month follow-up, the net benefit of predicting PFS event risk using the nomogram was higher for threshold probabilities between 0 and 0.680. At the 12-month follow-up, the net benefit was higher for threshold probabilities between 0 and 0.830 (Figure 3D).

## Discussion

In this prospective cohort study, we demonstrated that the inclusion of Huaier granules alongside standard targeted therapy and immunotherapy markedly improved progression-free survival in patients with unresectable hepatocellular carcinoma (HCC), extending median PFS from 5 months in the non-exposure group to 8.9 months in the exposure group (P = 0.001; HR = 0.50; 95% CI 0.32–0.78). This significant enhancement not only underscores the potential of Huaier granules as an effective adjunct in HCC treatment. Furthermore, our findings highlight a notable improvement in the quality of life, particularly in reducing fatigue, which is often one of the most debilitating symptoms for cancer patients, thereby supporting the integration of traditional Chinese medicine in modern oncologic care regimens. The primary bioactive constituents of Huaier consist of fungal-derived compounds, including polysaccharides, proteins, ketones, alkaloids, and minerals, which are part of the main essential nutrients required for the body to function and maintain overall health to avoid fatigue. From the TCM perspective, Huaier can also enhance Qi, invigorate blood, and resolve blood stasis, which make Qi and blood move through the body very well to help fight fatigue. Not only was quality of life improvement observed in our study as first-line treatment setting, but also it was shown the same as adjuvant therapy (Shi et al., 2023).

*In vitro* experiments and animal experiment data revealed the underlying anti-HCC mechanism of Huaier and further showed that Huaier had synergistic effect with targeted therapy of sorafenib. Li X. et al. (2024) observed that HP showed a weaker proliferation inhibitory effect on the mouse source and human HCC cells *in vitro* but exhibited stronger anti-HCC effects in animals. And nude mice models confirmed that macrophages play an important role in the anti-HCC effect of Huaier. Zhang et al. (2024) utilized 7-day-old goldfish embryos as hosts and successfully established an orthotopic xenograft model of HCC in goldfish livers. They evaluated the efficacy of the targeted therapy drug Sorafenib and Huaier granules, alone or in combination in the goldfish HCC orthotopic xenograft model and found that the combination therapy showed the best efficacy against HCC cells in terms of macrophage infiltration, polarization as well as tumor cells proliferation, metastasis and apoptosis.

In the subgroup analysis of patients with a history of alcohol consumption, the PFS of the exposure group was significantly extended to 14 months. This is related to the well-documented effect of moderate alcohol consumption in improving the tumor microenvironment, enhancing T-cell activation, and boosting immune responses (Bonavita et al., 2020). Huaier granules can upregulate PD-L1 expression in tumor tissues, positively modulating immune function (Li H. et al., 2024). Therefore, a history of alcohol consumption may enhance T-cell activation and improve immune responses, combined with the immunoregulatory effects of ICIs and Huaier granules, contributing to prolonged PFS. A similar trend was observed in the hepatitis subgroup, likely related to the role of inflammatory factors in improving the immune microenvironment. Vascular invasion is recognized as a critical marker of poor prognosis, representing greater invasiveness and metastatic potential, significantly shortening patient survival (Li et al., 2020). In this study, we found that in the subgroup with vascular invasion, patients treated with Huaier granules experienced a significant extension of PFS to 13 months. This effect may be related to Huaier granules' ability to inhibit tumor angiogenesis and suppress tumor metastasis (Li et al., 2020).

In China, most HCC cases occur against the background of viral hepatitis and cirrhosis. Statistics show that approximately 20% of hepatitis patients eventually develop cirrhosis, with nearly 80% potentially progressing to HCC (Rizzo et al., 2022). In our study, the subgroup analysis of cirrhosis patients demonstrated that combining Huaier granules extended PFS to 10 months. Huaier granules may exert their effects by enhancing Qi, invigorating blood, and resolving blood stasis. These actions can inhibit inflammatory factors and the epithelial-mesenchymal transition, thereby slowing the progression of cirrhosis (Luo et al., 2023). By delaying the progression of cirrhosis, liver function can be preserved, which in turn improves the tolerance of HCC patients to anti-tumor treatments, ultimately enhancing patient survival.

In this study, the treatment subgroup analysis included patients who received sintilimab combined with bevacizumab. The results indicate variable efficacy when sintilimab was part of the therapeutic regimen. For patients treated with sintilimab plus bevacizumab, the mPFS was 6.8 months, compared to 5 months in patients who did not receive this combination, although this difference did not reach statistical significance (P = 0.1488). In the subgroup receiving sintilimab alone, mPFS extended to 9 months, compared to 5 months for those not treated with sintilimab, suggesting a more favorable outcome, although this result approached but did not reach statistical significance (P = 0.0683). These findings suggest a potential benefit of sintilimab in extending PFS among patients with unresectable hepatocellular carcinoma when used in combination with other treatments, although the benefits vary depending on the combination of therapies used. Further studies are warranted to clarify the specific impact of sintilimab, particularly in combination regimens, to better understand its role in the management of hepatocellular carcinoma.

AFP was initially identified as a specific tumor marker in serum, later confirmed to exist in tumor cell cytoplasm, functioning as an oncogene (Chen et al., 2020). As a tumor marker, AFP assists in diagnosing HCC and predicting prognosis, but as an oncogene, it resists apoptosis, promotes proliferation, migration, and invasion of tumor cells (Zhang et al., 2020; Li H. et al., 2021), inhibits immune function, and facilitates immune evasion (Munson et al., 2023). In HCC treatment, AFP is a key cytokine leading to resistance against anti-tumor therapies. Studies have found that silencing the AFP gene can enhance the efficacy of treatments like all-trans retinoic acid analogs for HCC (Li W. et al., 2021). Based on the aforementioned reasons, the poor prognosis associated with high serum AFP levels is primarily due to its function as an oncogene that promotes tumor initiation, progression, and metastasis. Studies have shown that the traditional Chinese medicine, Huaier granules, can reduce the expression of serum AFP (Shi et al., 2024). In our previous research, we found that Huaier granules can downregulate AFP expression, leading to more significant benefits in patients with high AFP expression by inhibiting tumor growth.

Results indicate that extrahepatic metastasis is an independent prognostic factor, with a disease progression risk 1.807 times higher in patients with extrahepatic metastasis than those without (*HR =* 1.807, 95% CI 1.138–2.867, *P =* 0.013). The combination with Huaier granules was identified as a protective factor (*HR =* 0.514, 95% CI 0.326–0.810, *P =* 0.004). Therefore, patients with extrahepatic metastasis and without exposure to Huaier granules are more likely to experience disease progression. From the perspective of traditional Chinese medicine, according to the Pharmacopoeia, Huaier has a bitter taste and is non-toxic, functioning to “treat wind, break blood, and enhance strength,” promoting vital energy and blood circulation while eliminating tumors. It is thought that the core pathogenesis of liver cancer involves an underlying deficiency (Chang et al., 2023), with subsequent treatments like surgery, chemotherapy, radiotherapy, and targeted therapies further depleting vital energy. Vital energy deficiency persists throughout HCC, suggesting that herbal interventions should be integrated into liver cancer treatment, including post-surgery and in combination with localized or molecular-targeted therapies in intermediate and advanced stages. It has been confirmed that integrating Chinese herbal medicine can synergistically inhibit tumor growth and improve patients' quality of life (Wang et al., 2019). Basic research indicates that herbal medicine can upregulate immune responses, inhibit tumor growth and metastasis, and promote tumor cell apoptosis (Wang et al., 2020).

However, this study has several limitations that warrant careful consideration when interpreting its results. First, the follow-up period, concluding in October 2023, does not allow for a comprehensive assessment of long-term outcomes such as overall survival, suggesting a need for extended follow-up to more accurately evaluate the treatment’s efficacy. An extension phase of survival follow up is ongoing by collecting patients’ death events through phone call every 3 months. In addition, the lack of blinding for both patients and clinicians could introduce bias, particularly in subjective assessments such as quality of life. The use of diagnostic criteria based on Traditional Chinese Medicine, specifically Qi Deficiency and Blood Stasis Syndrome, may not resonate in non-TCM clinical settings. Future research will use blinding to mitigate and limit the occurrence of conscious and unconscious biases. Several relative points will be considered, including clinical supply planning, dispensing and inventory management, and disclosure of unplanned unblinding events. Lastly, despite efforts to balance baseline characteristics, the potential influence of unmeasured confounding factors, including lifestyle and genetic disparities, cannot be discounted.

In conclusion, the results of this study illustrate the significant clinical benefit of incorporating Huaier granules with standard targeted therapy and immunotherapy in the treatment of unresectable hepatocellular carcinoma. The use of Huaier granules led to a substantial extension of progression-free survival and was associated with notable improvements in the quality of life, specifically in managing fatigue. These findings suggest that Huaier granules can play a crucial role in enhancing the efficacy of existing cancer therapies while simultaneously managing symptoms, thereby providing a dual therapeutic benefit. Furthermore, the safety profile of this combination therapy proved to be favorable, encouraging its use in clinical practice. This study not only supports the incorporation of traditional Chinese medicine into contemporary treatment paradigms but also calls for further investigation into its mechanisms and potential benefits across different patient subgroups. Future research should focus on randomized, blinded, controlled trials to confirm these results and to explore the long-term outcomes and optimal integration of Huaier granules in the management of liver cancer.

## Data Availability

The raw data supporting the conclusions of this article will be made available by the authors, without undue reservation.

## Supplementary material

SUPPLEMENTARY FIGURE 1 Kaplan-Meier survival curves comparing clinical outcomes of exposed and non-exposed groups across various subgroups.
