# Supplementary figures and images for: Enhancing survival outcomes in unresectable hepatocellular carcinoma: a prospective cohort study on the effects of Huaier granules with targeted therapy plus immunotherapy
Source: Front Pharmacol. 2025 Mar 27;16:1529010. doi: 10.3389/fphar.2025.1529010 (PMC11983566; doi:10.3389/fphar.2025.1529010)

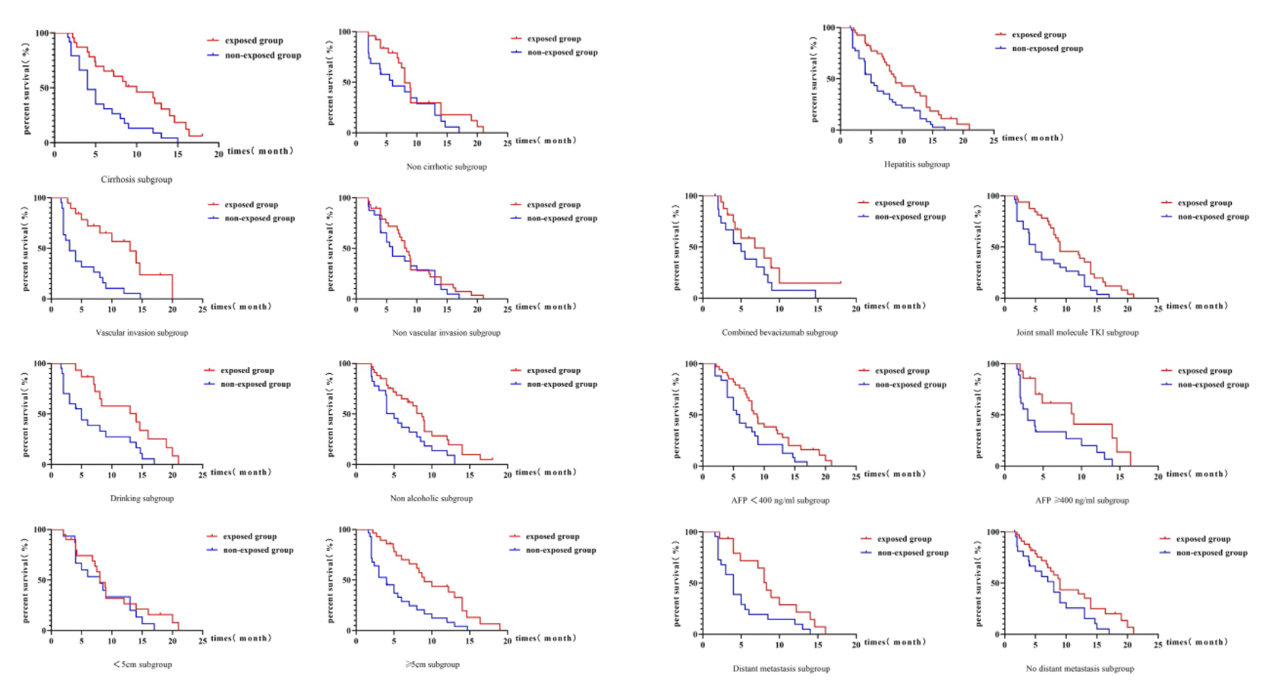

Supplement: Supplementary file 1 [file Image1.TIF]
